# Childhood trauma in a sample of patients with psychosis and healthy brothers

**DOI:** 10.1192/j.eurpsy.2021.563

**Published:** 2021-08-13

**Authors:** R. Hernandez Anton, G. Gutierrez Talavera, M. Zandio Zorrilla, L. Moreno Izco, A. Sánchez-Torres

**Affiliations:** PsiquiatrÍa, COMPLEJO HOSPITALARIO DE NAVARRA, PAMPLONA, Spain

**Keywords:** childhood trauma, CASH, CTQ, WHODAS

## Abstract

**Introduction:**

Psychosis are complex disorders due to their symptomatic and evolutionary heterogeneity. The genetic-environmental interaction model is the most accepted etiopathogenic model, in which neurobiological processes (genetic factors, connectivity and brain structure) and environmental factors (for example: childhood trauma) are studied. The association between suffering traumatic events in childhood and the subsequent development of a Mental Disorder is of increasing interest.

**Objectives:**

Analyze if a childhood trauma is a modulating factor of psychotic symptoms in patients with Mental Disorder. Analyze the implication of childhood trauma in long-term functionality.

**Methods:**

The sample is made up of 37 patients with psychosis and their healthy brothers. Different sociodemographic, clinical and evolutionary variables were collected in all groups. The sample was evaluated using the semi-structured interview CASH, the WHODAS scale and the self-applied questionnaire CTQ.

**Results:**

We did not find significant differences between the scores of CTQ between patients with psychosis and their healthy brothers. Sexual abuse is significantly correlated with the presence of hallucinations, inappropriate affect, formal thought disorders and catatonic symptoms. Emotional neglect is significantly correlated with the presence of hallucinations, inappropriate affect, affective blunting, and anhedonia. Physical neglect is significantly correlated with flattery and blunt affection. Sexual abuse is correlated with poorer personal care. Emotional neglect is correlated with poorer personal care, poorer family functioning, and worse overall functioning in the last year.

**Conclusions:**

The intensity of traumatic experiences throughout childhood could be considered a modulating factor of psychotic symptoms (positive, negative, disorganized and catatonic) and overall functioning (occupational, family, social and personal care).

